# Calcium Ion-Induced Structural Changes in Carboxymethylcellulose Solutions and Their Effects on Adsorption on Cellulose Surfaces

**DOI:** 10.1021/acs.biomac.1c00895

**Published:** 2021-12-22

**Authors:** Vishnu Arumughan, Tiina Nypelö, Merima Hasani, Anette Larsson

**Affiliations:** † Department of Chemistry and Chemical Engineering, Chalmers University of Technology, SE-412 96 Gothenburg, Sweden; ‡ AvanCell, Chalmers University of Technology, SE-412 96 Gothenburg, Sweden; § Wallenberg Wood Science Center, Chalmers University of Technology, SE-412 96 Gothenburg, Sweden; ∥ FibRe—Centre for Lignocellulose-based Thermoplastics, Department of Chemistry and Chemical Engineering, Chalmers University of Technology, SE-412 96 Gothenburg, Sweden

## Abstract

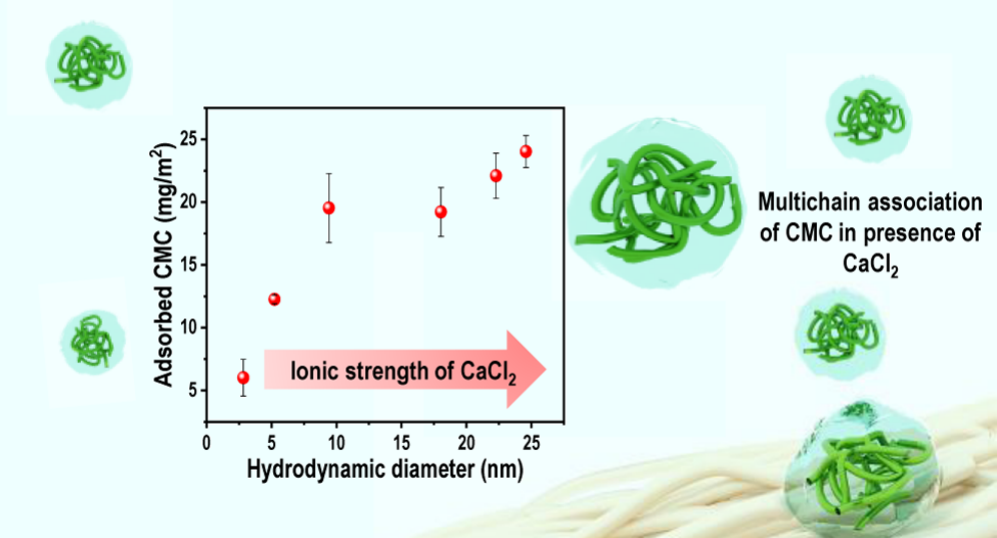

The adsorption of carboxymethylcellulose (CMC) on cellulose surfaces is one of the most studied examples of the adsorption of an anionic polyelectrolyte on a like-charged surface. It has been suggested that divalent ions can act as a bridge between CMC chains and the surface of cellulose and enhance the CMC adsorption: they can, however, also alter the structure of CMCs in the solution. In previous investigations, the influence of cations on solution properties has been largely overlooked. This study investigates the effect of Ca^2+^ ions on the properties of CMC solutions as well as the influence on cellulose nanofibers (CNFs), which was studied by dynamic light scattering and correlated with the adsorption of CMC on a cellulose surface probed using QCM-D. The presence of Ca^2+^ facilitated the multichain association of CMC chains and increased the hydrodynamic diameter. This suggests that the adsorption of CMCs at high concentrations of CaCl_2_ is governed mainly by changes in solution properties rather than by changes in the cellulose surface. Furthermore, an entropy-driven mechanism has been suggested for the adsorption of CMC on cellulose. By comparing the adsorption of CMC from H_2_O and D_2_O, it was found that the release of water from the cellulose surface is driving the adsorption of CMC.

## 1 Introduction

The adsorption of polymers on cellulose surfaces has been of interest for decades due to its applications in papermaking. The rapid development of layer-by-layer surface modification techniques on cellulose materials vitalized further the need for understanding polymer adsorption on cellulose.^1,2^ Cationic polyelectrolytes readily adsorb on negatively charged cellulose surfaces, with interactions governing entropy gain due to the release of counterions.^3^ It has been shown that anionic polyelectrolytes such as carboxymethylcellulose can also adsorb on cellulose surfaces irrespective of their expected electrostatic repulsion.^4−8^ Carboxymethylcellulose (CMC) is the most versatile semisynthetic polyelectrolyte derived from cellulose^9,10^ and has been used in a wide range of applications, including mineral processing,^11−13^ pharmaceutics,^14^ and as a viscosity modifier.^15^ In the paper industry, the adsorption of CMC on cellulose has been used to improve the wet mechanical properties of cellulose.^4,5,16^ Apart from conventional applications, the adsorption of CMC on cellulose has been used to create biofunctional interfaces for diagnostic platforms and bone-healing scaffolds.^6,17,18^

Seminal work done by Laine et al. suggested a cocrystallization mechanism due to the similar backbone structure of CMC and cellulose.^4^ However, the mechanism of adsorption and the nature of the driving force behind the interaction of CMC with cellulose surface are still ambiguous. The adsorption of CMC on cellulose surfaces has been probed using surface-sensitive techniques, such as QCM-D and SPR, to distinguish the controlling factors of the adsorption of CMCs on cellulose fibers.^7,8^ Kargl et al. studied the adsorption of CMC on surfaces of varying cellulose character and suggested the involvement of a specific interaction between the CMC chains and the cellulose surface:^7^ interactions between them can be modulated by changing the concentration of the electrolyte in the system.^4,8^ The valency of cations also plays a critical role in the adsorption process: it has been shown that the presence of Ca^2+^ ions enhanced CMC adsorption and that the adsorbed layer was stable compared to that formed in the presence of Na^+^ ions. Taking these observations into consideration, it has been suggested that Ca^2+^ ions act as a bridge between a negatively charged CMC and a cellulose surface.^8^

Adsorption is a multifaceted interfacial process where characteristics of both the polymer solution and the adsorbing surface are important. Recent computational studies suggest that the adsorption of anionic polyelectrolytes on like-charged surfaces is driven by multivalent ion-induced charge inversion, either on adsorbing surface or in polyelectrolytes.^19,20^ This has been observed experimentally by Tiraferri et al. for the case of adsorption of negatively charged polystyrene sulfonate on a silica substrate.^21^ A recent investigation by Jansson et al. revealed multivalent ion-induced charge inversion in clay nanoplatelets.^22^ However, such multivalent ion-induced charge inversion has not yet been reported in the case of cellulose-based materials. The multivalent ions were shown to screen the surface charge to a large extent and induce interactions between carboxylated cellulose nanoparticles through a ion–ion correlation and dispersion interactions.^23^ Recent studies on dilute CMCs solutions have shown that the interaction of polyelectrolytes with multivalent ions can affect the conformation of polymer chains in solution, supramolecular association, solubility, and flow behavior.^24,25^ The addition of salt typically reduces the viscosity of polyelectrolyte solutions due to the reduced expansion of polymer chains in the solution. This has been observed in CMC in the presence of NaCl. On the other hand, divalent ions increased the viscosity of CMC solutions significantly; it has been suggested that multivalent ions cross-link the CMC chain via electrostatic bridging and that this cross-linked network contributes to the viscosity.^25^ Although Sharratt et al. observed the formation of 20–40 nm clusters of CMC chains in the presence of multivalent ions,^24^ the influence of these structural changes in solutions on interfacial processes such as adsorption has not yet been studied systematically.

The focus of the present investigation is to understand the adsorption process in the presence of Ca^2+^ ions by considering structural changes that occur in CMC solutions as well as changes in the cellulose surface in terms of charge. Furthermore, the adsorption of CMC from deuterated water and water is compared to test the emerging hypothesis that the adsorption of nonionic and anionic polymeric systems is driven by release of structured water from the cellulose surface.^26−28^ Considering the ongoing interest in integrating CMC adsorption with the current pulping process to modify fibers within the production line,^29^ knowledge of the multivalent ion-induced effect in CMC adsorption would be useful in designing the unit operations of the industrial process. Moreover, the outcome of this research contributes to the fundamental understanding of the adsorption of anionic polyelectrolytes on like-charged surfaces.

## 2 Experimental Section

### 2.1 Materials

The cellulose nanofibers (CNF) with an average diameter of 5 nm and carboxylic content of 31.4 μmol/g from softwood Kraft fibers were obtained from Stora Enso, Stockholm. The nanocellulose fibers have a residual hemicellulose content of 14.7% (xylose 8%, arabinose 0.62%, galactose 0.25%, and mannose 6.1%) and lignin content of 1.1% (Klason lignin 0.35% and acid-soluble lignin 0.75%). Calcium chloride (CaCl_2_), deuterium oxide (D_2_O), and polyethyleneimine-branched polymers (with an average Mw 25 kDa) were purchased from Sigma-Aldrich. Carboxymethylcellulose (CMC), more precisely Blanose 7LPEP with a molecular weight of 90.5 kDa and a degree of substitution (DS) of 0.7 (according to the supplier), was kindly provided by Ashland.

### 2.2 Methods

#### 2.2.1 Preparation and Characterization of an Ultrathin Cellulose Model Film for Adsorption Studies

A cellulose model film was prepared according to the protocol described in our previous report.^30^ A CNF suspension of concentration 1.6 g/L was sonicated and centrifuged; the supernatant-containing CNFs were spin-coated on SiO_2_-coated QCM-D sensors supplied by Q-Sense AB (Gothenburg, Sweden). Prior to the spin coating with CNF, the sensors were preabsorbed with an anchoring layer of polyethyleneimine to promote the CNF attachment and film stability. The spin-coated QCM sensors were dried in an oven at 80 °C for 10 min and stored in a desiccator.

The film morphology and uniformity were analyzed using atomic force microscopy (INTEGRA Prima setup NT-MDT Spectrum Instruments, Moscow, Russia). The height profiles of three random spots on the film were recorded in semicontact mode, and the root-mean-square roughness was calculated using Gwyddion software to assess the quality of the film.

The water content of the CNF model film was calculated according to the procedure reported by Kittle et al.^30^ using a QCM-D instrument (Biolin Scientific, Gothenburg, Sweden). In this procedure, the QCM-D sensors coated with CNFs were placed in the flow cell, and deionized water was injected into the flow cell at the rate of 100 μL per minute for 3 h to get a stable baseline. Then, the solvent was switched into D_2_O, and an immediate drop in frequency was observed due to the relatively high density of D_2_O. After 10 min, the solvent was again switched to water, and the shift in the frequency was recorded. The difference in the frequency changes for the bare sensor, and the CNF-coated sensor during H_2_O–D_2_O exchange was used for the calculation of water associated with the spin-coated CNF film.

#### 2.2.2 Hydrodynamic Size of CMCs Determined by Dynamic Light Scattering

The hydrodynamic size of CMCs in different electrolyte concentrations was determined using DLS (Zetasizer Nano Zs, Malvern Instruments, U.K.). CMC solutions with a concentration of 0.2% (w/v) were prepared by dissolving CMC in Milli-Q water and stirring overnight to ensure complete dissolution. The electrolyte concentration in the solution was adjusted by adding 1 M CaCl_2_ solution filtered with a 0.45 μm filter. Disposable plastic cuvettes were used for DLS measurements, repeated three times with 25 runs per measurement.

#### 2.2.3 ζ Potential and Electrophoretic Mobility Studies

The effect of Ca^2+^ ion concentration on electrophoretic mobility and the ζ potential of CMC solutions and CNF (in suspension) were analyzed using Zetasizer Nano Zs (Malvern instruments U.K.). A 0.5% (w/v) CNF suspension was probe sonicated in an ice bath for 1 min and diluted 10 times to get a CNF suspension of concentration 0.05% (w/v); the electrolyte concentration of the suspension was adjusted by adding an appropriated amount of 1 M CaCl_2_. The CMC solutions prepared for DLS measurements were used for electrophoretic mobility measurements.

#### 2.2.4 Adsorption Experiments Using QCM-D

The adsorption of CMC onto the cellulose surface from the solutions of varying concentrations of CaCl_2_ ranging from 5 to 250 mM was studied using QCM-D equipment (Biolin Scientific, Gothenburg, Sweden). All of the experiments were performed at 25 °C with a flow rate of 20 μL/min. A concentration of 0.2% (w/v) CMC solutions containing different concentrations of CaCl_2_ were injected into flow cells. The CNF film was equilibrated with CaCl_2_ solutions of corresponding concentrations prior to the injection of the CMC solution.

Similarly, adsorption of CMC on cellulose at 250 mM ionic strength of CaCl_2_ has been performed from D_2_O and H_2_O. A model introduced by Johannsmann et al. was used to calculate the adsorbed mass.^31^ According to Johannsmann, the shift in the complex frequency is related to the resonance frequency of the crystal in solution by the following equation.
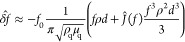
1where δ̂_f_ is the shift in the complex frequency, *f*_0_ is the fundamental resonance frequency of the quartz crystal in air, *f* is the resonance frequency of the crystal in contact with the solution, *d* is the thickness of the film, and  is the complex shear compliance. ρ_q_ and μ_q_ are the specific density and elastic shear modulus of the quartz crystal. Equation 3 can be written in a simpler form using equivalent mass (m*), which is defined as
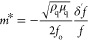
2Then, we obtain a linear equation
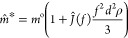
3It is assumed that ^*J*(*f*) is independent of the frequency in the accessible range and the true sensed mass *m*^o^ is obtained graphically by plotting equivalent mass against the square of the resonance frequency. In this investigation, third, fifth, and seventh overtones were used for Johannsmann’s modeling. It is important to mention that the true sensed mass calculated using Johannsmann’s modeling includes the mass of water that is associated with the adsorbed layer and thus not equal to the dry mass of adsorbed CMC.

#### 2.2.5 Swelling and Deswelling Studies Using QCM-D

The adsorbed layers of CMC were subjected to swelling by introducing distilled water into the flow cell with a flow rate of 20 μL/min. It is important that the flow rate be kept small to prevent flow-induced desorption. The swollen CMC layers were subjected to deswelling by the introduction of calcium chloride solution into the flow cell.

## 3 Results and Discussion

### 3.1 Characterization of the CNF Model Film

**Figure 1:**
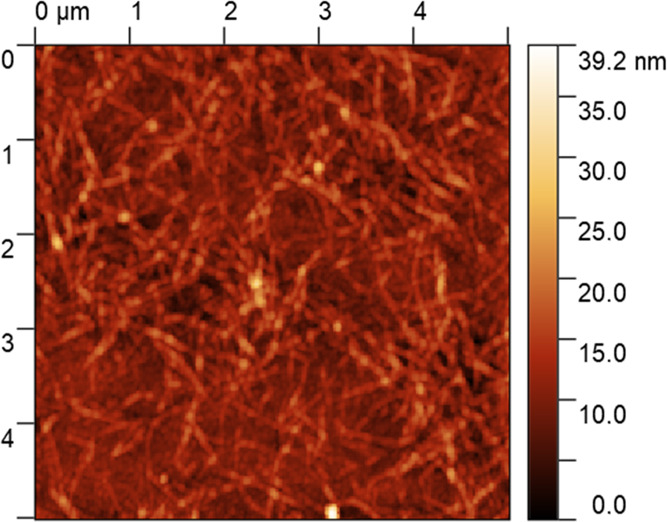
AFM height image of the CNF model film.

The spin coating of CNF on QCM-D sensors resulted in an ultrathin film with the fibrillar morphology illustrated in the AFM height image (Figure 1). The film showed a root-mean square roughness of 3.2 nm, determined using an area of 5 μm × 5 μm (Figure 1).

Immersion of the film into water, followed by solvent exchange to D_2_O, and then using the 10% mass difference of H_2_O and D_2_O to determine the water content of the film^30,32^ revealed that the CNF film is highly hydrated (41 mg m^–2^, see the Supporting Information S1). The solvent fraction estimated in the film is considered to be an indirect measure of the void volume on the surface of the CNF model film.^33^ Both AFM and solvent exchange studies revealed that the CNF film has a morphology that contributes to a large surface area that is available for interaction with the solvent and the adsorbates.

### 3.2 Carboxymethylcellulose in CaCl_2_ Solutions

The CMC solutions (0.2%w/v) were visually transparent; no phase separation could be observed in any of the concentrations of CaCl_2_ used in this study (5–250 mM). These results were contradictory to the observations made by Sharratt et al.,^24^ who showed that CMC shows an L-type phase behavior, i.e., a separate phase in the presence of very small concentrations of specifically interacting cations such as Ca^2+^ and Ba^2+^. The critical concentration of salt required to induce phase separation depends on the types of CMC (Mw and DS), salt, and the concentration of CMC.^34−36^ The CMC used in the present study has a significantly lower Mw (95 kDa) and a lower DS than the CMC used by Sharratt et al. (250 kDa),^24^ which can explain the difference observed in the phase behavior.

**Figure 2:**
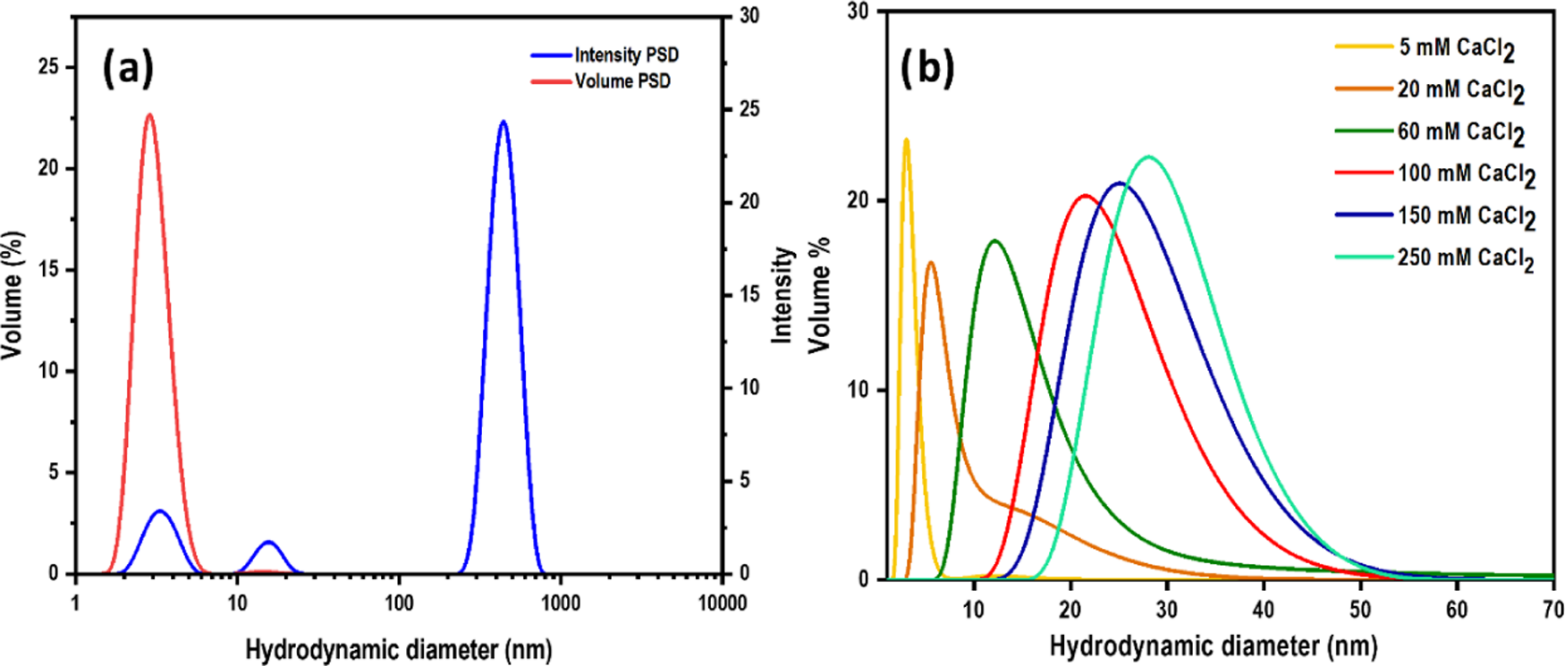
(a) Volume (red) and intensity (blue) PSD of CMC in 5 mM CaCl_2_. (b) Volume PSDs of the CMC for different ionic strengths of CaCl_2_.

The hydrodynamic diameters of the CMC solutions were determined using dynamic light scattering. The primary particle size distribution (PSD) obtained from the light scattering experiment is intensity PSD (red curve in Figure 2a), showing that the majority of the CMC has a hydrodynamic diameter exceeding 100 nm. In contrast, the volume PSD shows a single sharp peak, indicating that the majority of the CMC volume has a hydrodynamic diameter within 5 nm (Figure 2b). The contrasting information of the hydrodynamic size obtained from the intensity PSD and volume PSD of the same solution is due to the method used for extracting the hydrodynamic diameter from the autocorrelation function. The calculation of intensity PSD relies solely on the intensity of the light scattered by the solution. The larger particles dominate the scattering and, accordingly, they affect the particle size distribution more. Mie theory was used in the calculation of volume PSD, where optical properties such as the refractive index and the absorption of light by the material are considered together with the scattered intensity.^37^ Only the volume PSD will be used in the discussion henceforth since this is the standard procedure for reporting hydrodynamic size.

**Figure 3:**
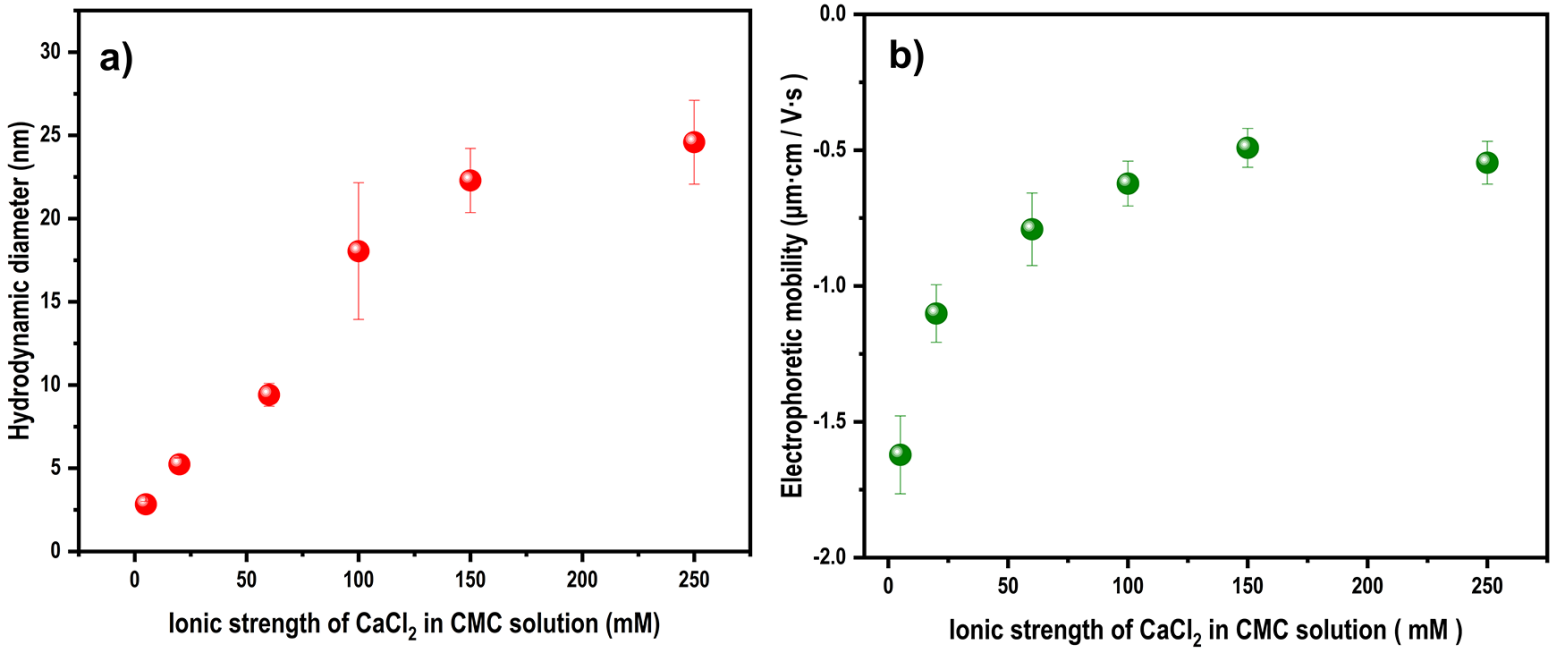
(a) Average hydrodynamic diameter obtained from the volume PSD and (b) electrophoretic mobility of CMC solutions for different ionic strengths of CaCl_2_.

Figure 2b represents the volume PSD of CMC solutions in 5–250 mM concentration of CaCl_2_. The increase in the concentration of Ca^2+^ ions in the solutions resulted in a larger average size and wider size distributions. The average size obtained from the major peak in the volume PSD is plotted as a function of the CaCl_2_ concentration (Figure 3a).

A linear increase in hydrodynamic size can be observed from 5 to 100 mM CaCl_2_. The curve levels off after 100 mM and, at 250 mM CaCl_2_, the average hydrodynamic diameter was around 25 nm. This concurs with the observation of Sharratt et al.^24^

The reason for the trend observed in the hydrodynamic diameter can be attributed to the interaction of Ca^2+^ with CMC polymer chains. It has been suggested that Ca^2+^ ions interact with CMC chains both through ion–ion correlations and dispersion interactions.^38^ This could reduce the charge density of CMCs close to neutral, as is evident from the electrophoretic mobility studies shown in Figure 3b. The electrophoretic mobility shifted to less negative values as the concentration of CaCl_2_ increased and started to level off from 60 mM. The reduction in the charge of CMC chains can promote both inter- and intrachain associations and the formation of multichain clusters, thereby leading to the trend observed for the hydrodynamic diameter of the CMC in different CaCl_2_ concentrations.

### 3.3 CNF Suspensions in Different Concentrations of CaCl_2_

**Figure 4:**
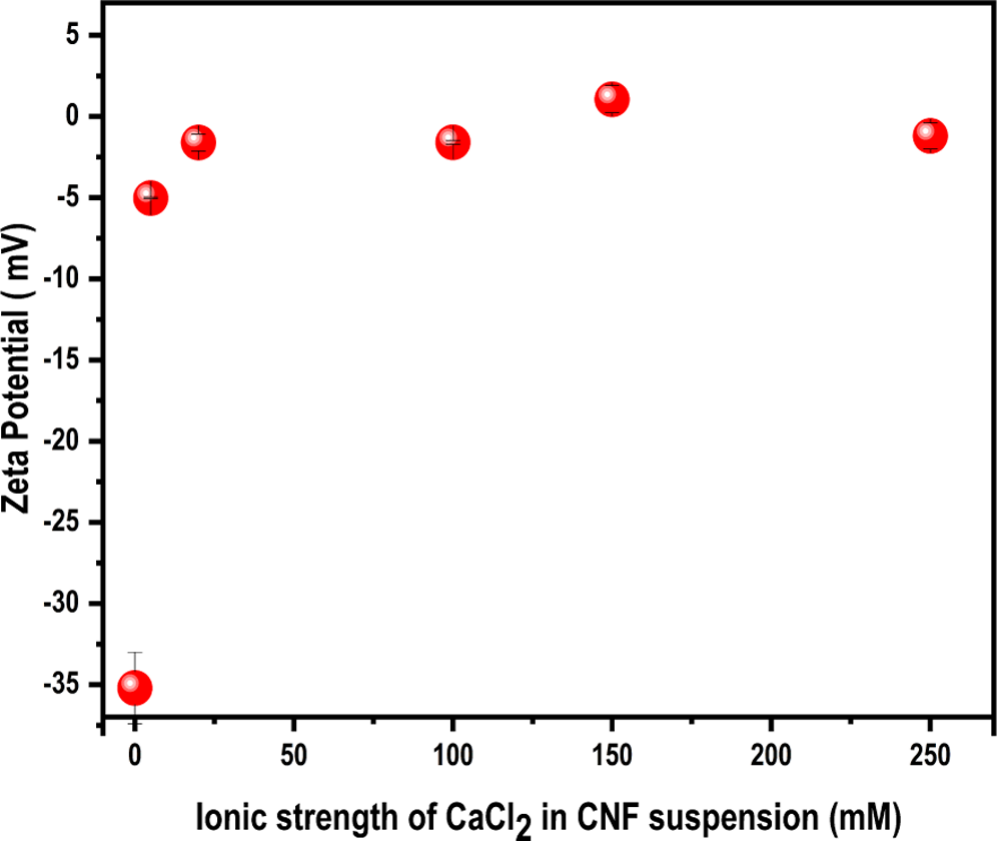
ζ Potential of CNF suspensions in different ionic strengths of CaCl_2_.

The effect of the concentration of CaCl_2_ on a cellulose surface was studied by analyzing the ζ potential of CNF suspensions (Figure 4). Ideally, this should have been done on CNF-coated QCM-D sensors: due to the experimental limitations of this study, it was performed on CNF suspensions instead. In CNF suspensions, there is a possibility of structural reorganization of CNFs in the presence of divalent cations. Valencia et al. have reported re-entrant transitions in the microstructure of TEMPO-oxidized CNF suspensions in the presence of divalent ions.^39^ A similar reorganization caused by cations on the CNF microstructure within a film might be more limited compared to observed for dispersions.

The CNF suspensions showed a ζ potential of −35 mV before adding CaCl_2_, indicating that the CNF surface was negatively charged and the suspension colloidally stable.^40,41^ The addition of 5 mM CaCl_2_ destabilized the colloidal suspension of CNF and resulted in a slightly turbid suspension. It should be kept in mind that the CNF suspensions were not colloidally stable in all of the concentrations of CaCl_2_ used: the ζ potential values obtained and presented in Figure 4, therefore, manifest not only the ζ potential but also the instability and should therefore only be considered as being qualitative in later discussions. However, the precipitation observed of CNFs indicates that surface charges are screened to a large extent, even at low concentrations of CaCl_2_.

### 3.4 Adsorption of CMC from CaCl_2_ Solutions

**Figure 5:**
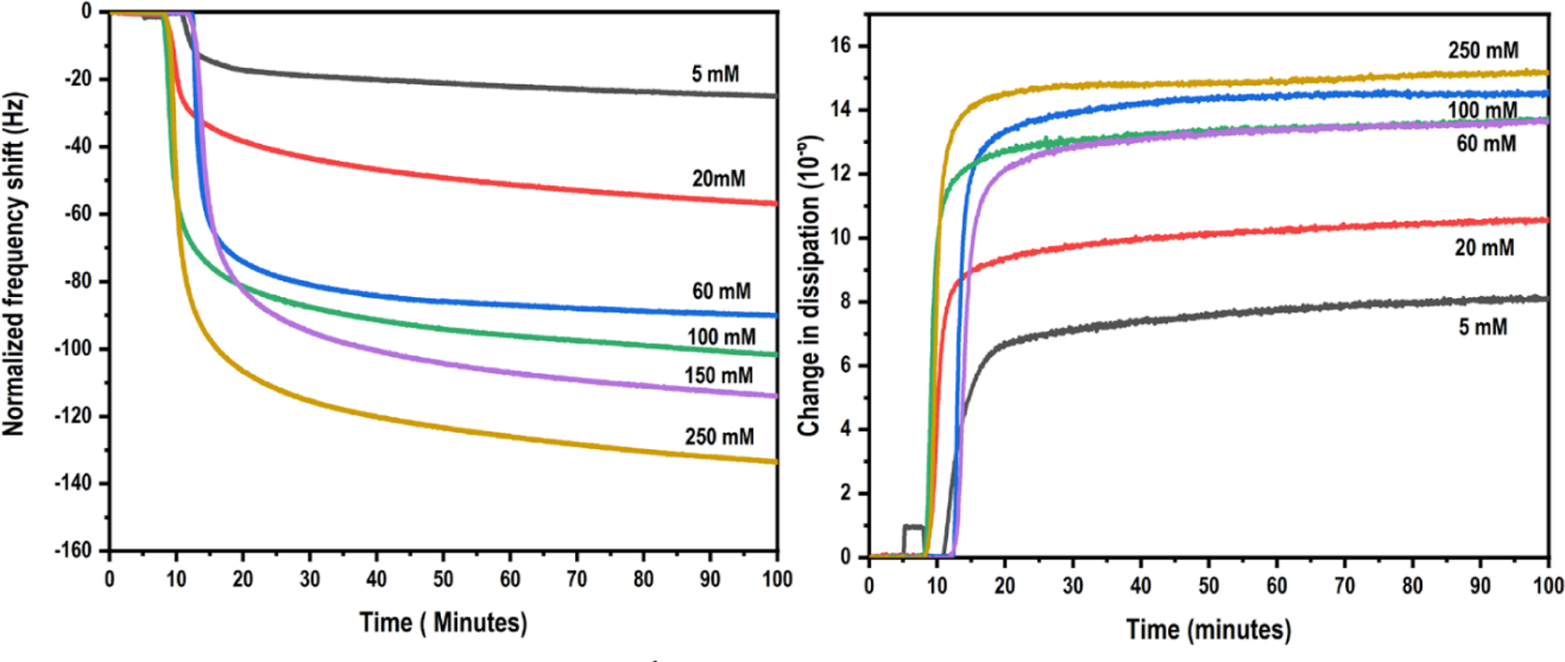
Representative (a) frequency (third overtone) and (b) dissipation curves for the adsorption of CMC on CNF model surfaces in aqueous CaCl_2_ environments.

The QCM-D technique enables real-time investigation of the adsorption process to be made in terms of the kinetics of adsorption, the viscoelastic nature of the adsorbed layers, and the mass adsorbed per unit area. Figure 5 shows representative QCM frequency and dissipation curves for CMC adsorption in the presence of CaCl_2_.

**Figure 6:**
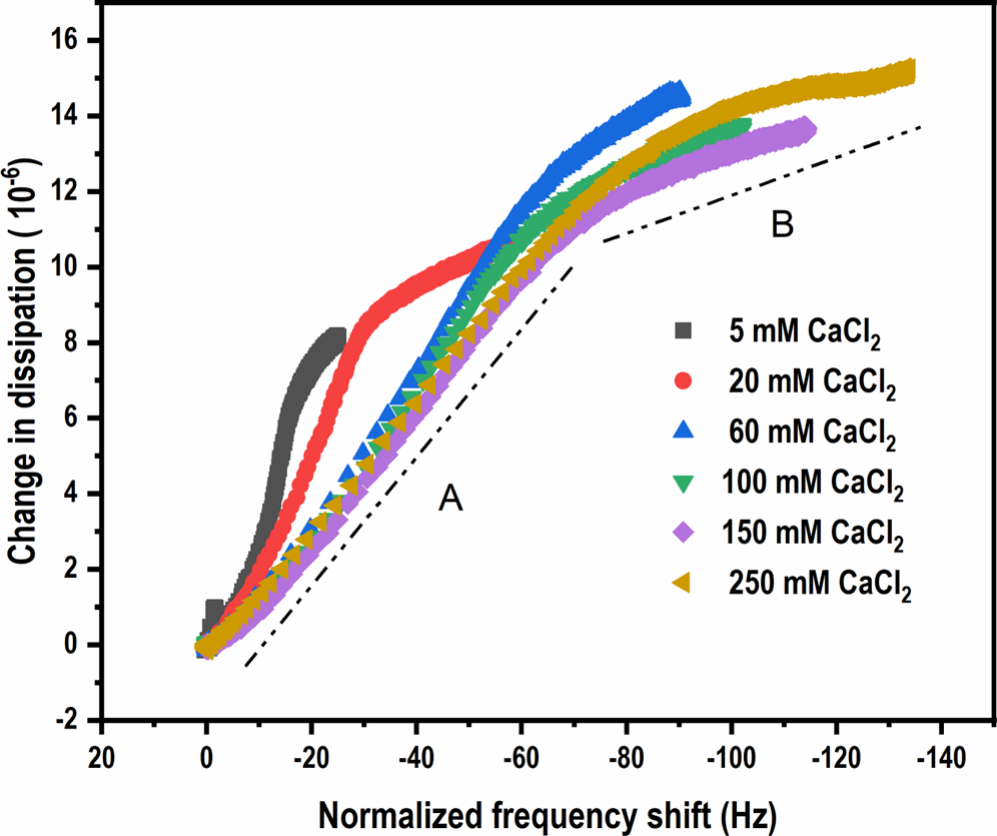
Change in dissipation as a function of normalized frequency shift (third overtone) during the adsorption of CMC on CNF model surfaces in aqueous CaCl_2_ environments.

Some general conclusions can be drawn from these adsorption curves: first, the adsorption process of CMCs onto the CNF model film is slow for all concentrations of CaCl_2_ studied, with the adsorption not reaching a plateau even after 90 min. The change in frequency and dissipation shift increase with the increasing concentrations of CaCl_2_, clearly showing that the CaCl_2_ concentration has a profound effect on the CMC adsorption process. This is in accordance with observations made by Laine et al.^4^ and Liu et al.,^8^ who studied the adsorption of CMC onto cellulose-rich macrofibers and regenerated cellulose surfaces, respectively. The high dissipation values indicate that the adsorbed layer is viscoelastic. Further structural information on adsorbed CMC layers can be obtained by analyzing the so-called D–f plots, which represent the changes in the conformation of the adsorbed layer qualitatively as adsorption proceeds. Figure 6 shows representative D–f plots of the adsorption of CMC in different concentrations of CaCl_2._

D–f plots of CMC adsorption show a continuous curve in all of the CaCl_2_ concentrations studied, further confirming that the adsorption of CMC is a slow process.^42^ The increase in the slope of the D–f curves at lower concentrations of CaCl_2_ suggests that the adsorbed layer is more viscous, which is indicated by the increase in energy dissipation. An increase in the CaCl_2_ concentration, on the other hand, decreases the slope and hence the viscous component, resulting in the adsorbed CMC layer becoming denser.

**Figure 7:**
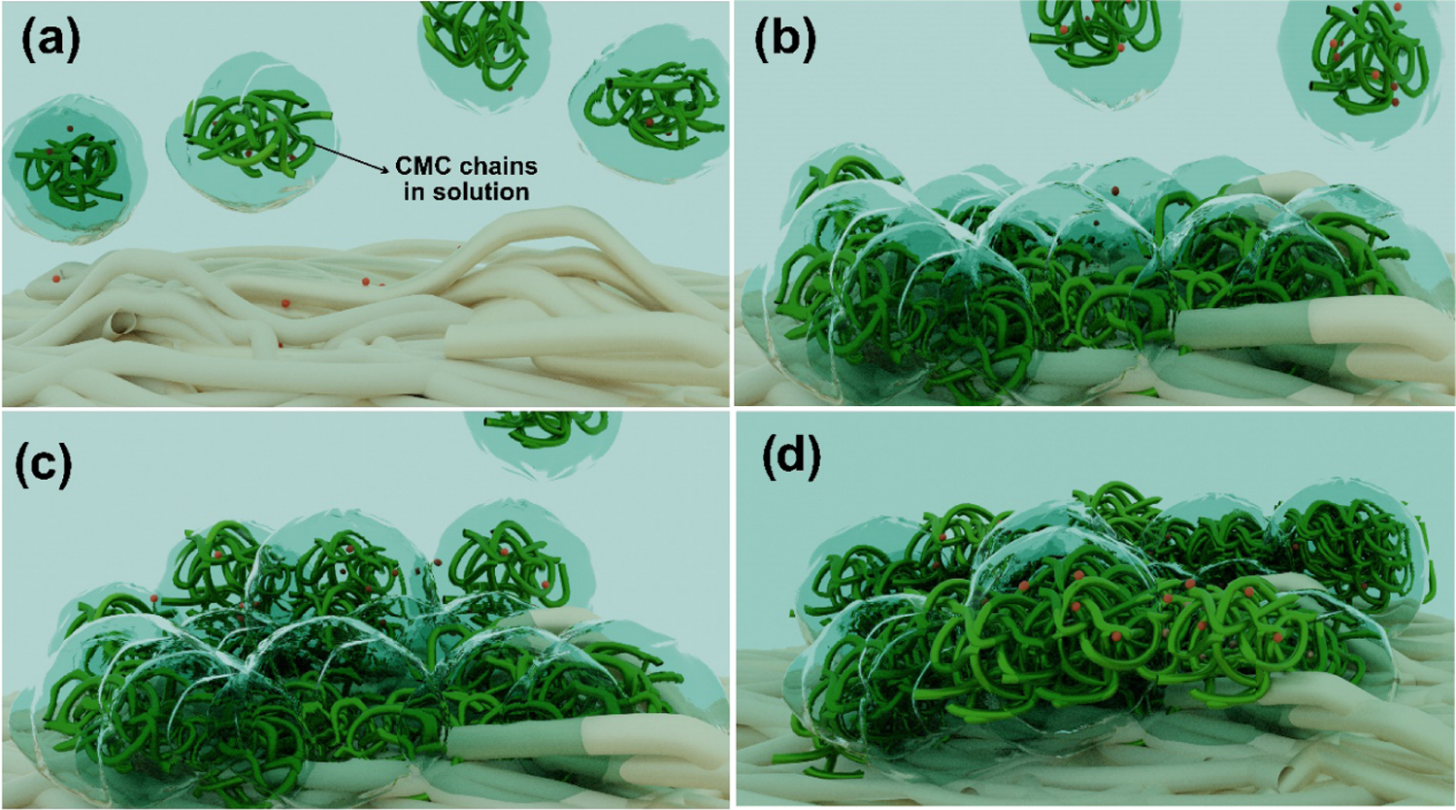
Schematic representation of CMC adsorption in the presence of CaCl_2_ (orange dots). (a) CMC chains (green) approach the surface. (b) Collapse and spread of CMC chains on the cellulose surface and into the pores of the CNF film. (c) Dangling CMC chains interact with adsorbed CMC on the cellulose surface (second stage) and desorption of shorter chains. (d) Dangling CMC chains penetrate the adsorbed CMC network to form a thicker adsorbed layer.

The degree of collapse of the polymer structure upon adsorption depends on the conformation of the polymers in solution (Figure 7a) and the strength of the interaction of the polymer with the surface.^43^ The denser layer formed at higher concentrations of CaCl_2_ indicates that the structure of polymer molecules that come into contact with the surface collapse (Figure 7b), which is also an indication of attractive interactions between the CMC and the cellulose surface. Kargl et al. suggested that CMC has a specific interaction with a cellulose surface,^7^ although the nature of this interaction has not yet been identified. The conformational rearrangement and spreading over the surface could be hampered kinetically by the adsorption of neighboring molecules. Consequently, some of the adsorbed CMC might not be directly involved in the immediate interaction with the cellulose surface (Figure 7c), which contributes to increasing the viscosity of the layer and hence the dissipation of energy. As the adsorption proceeds, the D–f curves (Figure 6) show a nonlinear adsorption behavior with a slope change, revealing that the conformation of the adsorbed CMC layer changes during adsorption. Similar observations have been reported by Köhnke et al. with respect to the adsorption of arabinoxylans on cellulose.^42^ The lower slope of region B in the D–f plot (Figure 6) indicates that dangling CMC layers diffuse toward the surface and transform the adsorbed layer, making them denser. It could also be arising due to conformational changes in the adsorbed layer due to the exchange of shorter adsorbed chains with larger CMC chains, which is thermodynamically favored.

It is possible that Ca^2+^ ions can still induce multichain association on the surface and, at this stage, the adsorbed layer can be considered as being a Ca^2+^ cross-linked gel network that has been attached to the cellulose surface.

**Figure 8:**
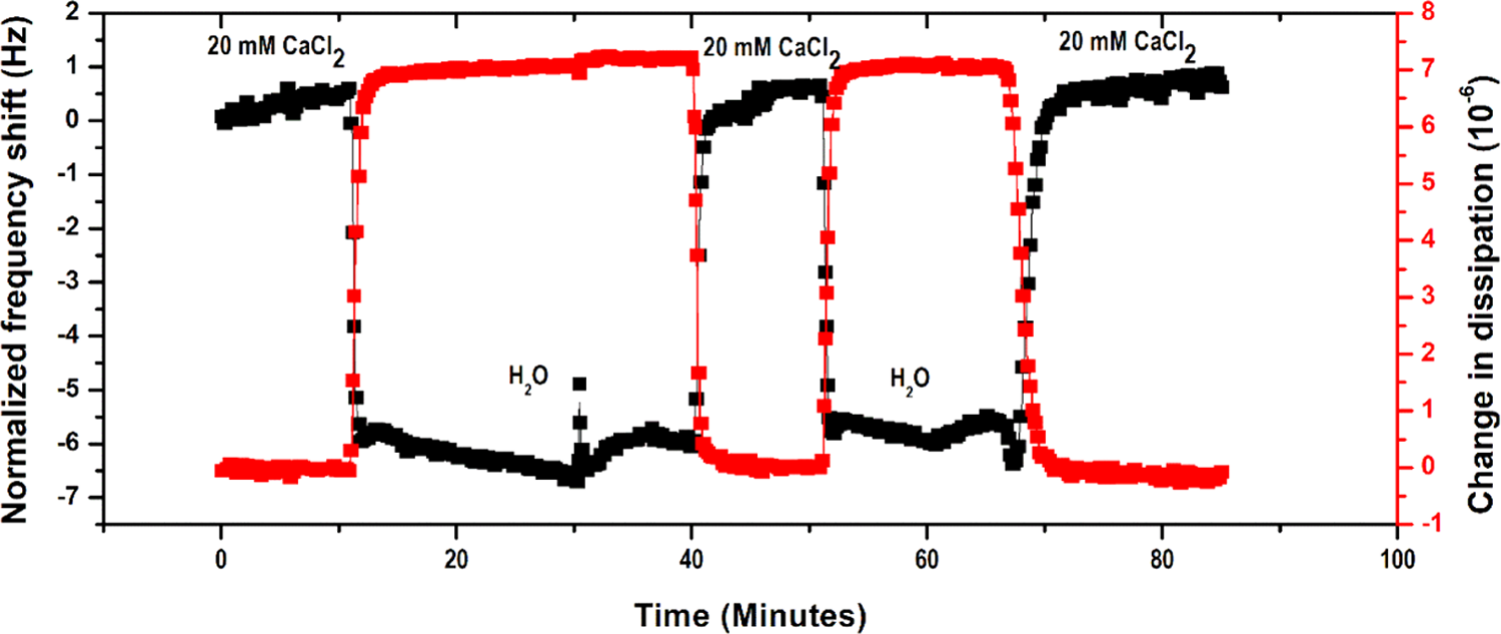
Swelling and deswelling cycles of the adsorbed layers of CMC on a cellulose surface.

The swelling and deswelling behavior of the adsorbed CMC layers in the presence of deionized water and 20 mM CaCl_2_ is illustrated in Figure 8. An immediate drop in frequency is observed when 20 mM CaCl_2_ is exchanged with deionized water. This corresponds to the water intake, which can also be termed as swelling of the adsorbed layer. Further introduction of 20 mM CaCl_2_ into the flow cell at 40 and 68 min increased in frequency, showing that the CMC layer deswells or collapses in the presence of Ca^2+^. Similar increases in frequency upon water injection into the CMC adsorbed film have been reported by Kargl et al.^7^ The swelling–deswelling behavior was reversible, two cycles of which are presented in Figure 8, thereby confirming the gel network nature of the adsorbed layers.

**Figure 9:**
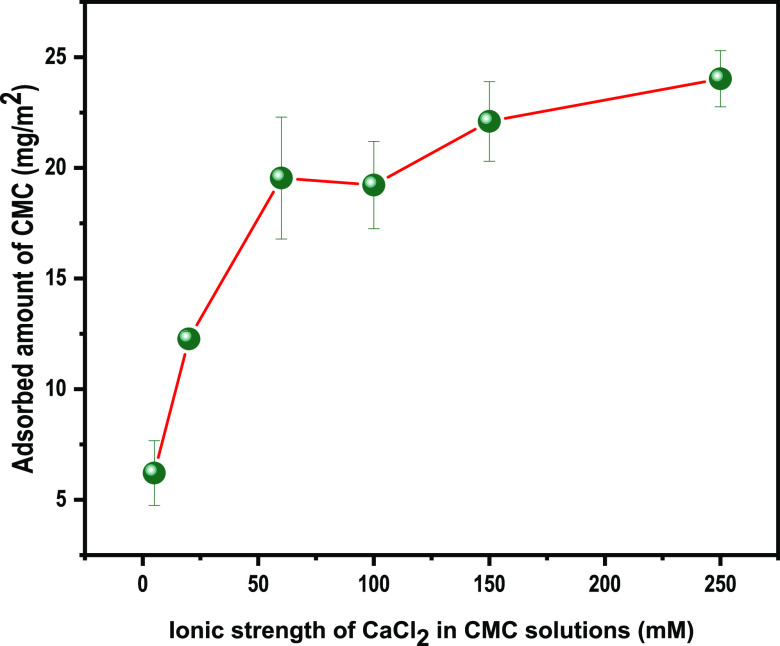
Mass of the adsorbed CMC per unit area on a cellulose surface from CMC solutions with different concentrations of CaCl_2_.

The adsorbed CMC per unit area, calculated using Johannsmann’s model, is plotted against the concentration of CaCl_2,_ as shown in Figure 9.

Adsorption increases as the concentration of CaCl_2_ increases, which agrees with the observations of Laine et al. and Liu et al.^4,8^ The mass of the CMC adsorbed per unit area increased significantly from 5 to 60 mM CaCl_2_ and started to level off. The ionic strength 5 mM corresponds to a Ca^2+^ to CMC charge ratio of 4. (The properties of the CMC solution has also been expressed in terms of the charge ratio between Ca^2+^ and CMC, which can be found in the Supporting Information.) As can be seen in Figure 9, the shape of this adsorption isotherm is somewhat similar to the isotherm for polyelectrolytes adsorbed on like-charged surfaces, as predicted by Scheutjens–Fleer polymer adsorption theory (S-F theory). In this theory, polymer adsorption is described in terms of four parameters: **χ**, ***χ***_**s**_,***q***_**m**_, and **σ**_**0**_, where **χ** and **χ**_**s**_ are the Flory–Huggins parameters accounting for polymer–solvent interactions and the polymer segment-surface interaction, respectively.^44^ Both **χ** and **χ**_**s**_ have a positive linear dependence on the adsorbed amount. The terms ***q***_**m**_ and **σ**_**0**_ represent the surface charge and polymer segmental charge, respectively, and account for Columbic interactions in polyelectrolyte adsorption.^44−46^ According to S-F theory, the charges present in both the polyelectrolytes and the surface at higher salt concentrations are screened (*i.e., **q***_**m**_ and **σ**_**0**_), and the adsorption will be purely governed by nonelectrostatic forces, which is accounted for by **χ**_**s**_. It is noteworthy that S-F theory only considers monovalent salts. However, a comparison of the ability of divalent ions with monovalent ions to increase the adsorbed amount at the same ionic strength suggests that, apart from obvious electrostatic screening effects, divalent metal ions contribute positively to the adsorption process.^8^ This positive contribution of multivalent ions to adsorption might originate from the structural changes in polymer solutions induced by multivalent ions.^25,47^

**Figure 10:**
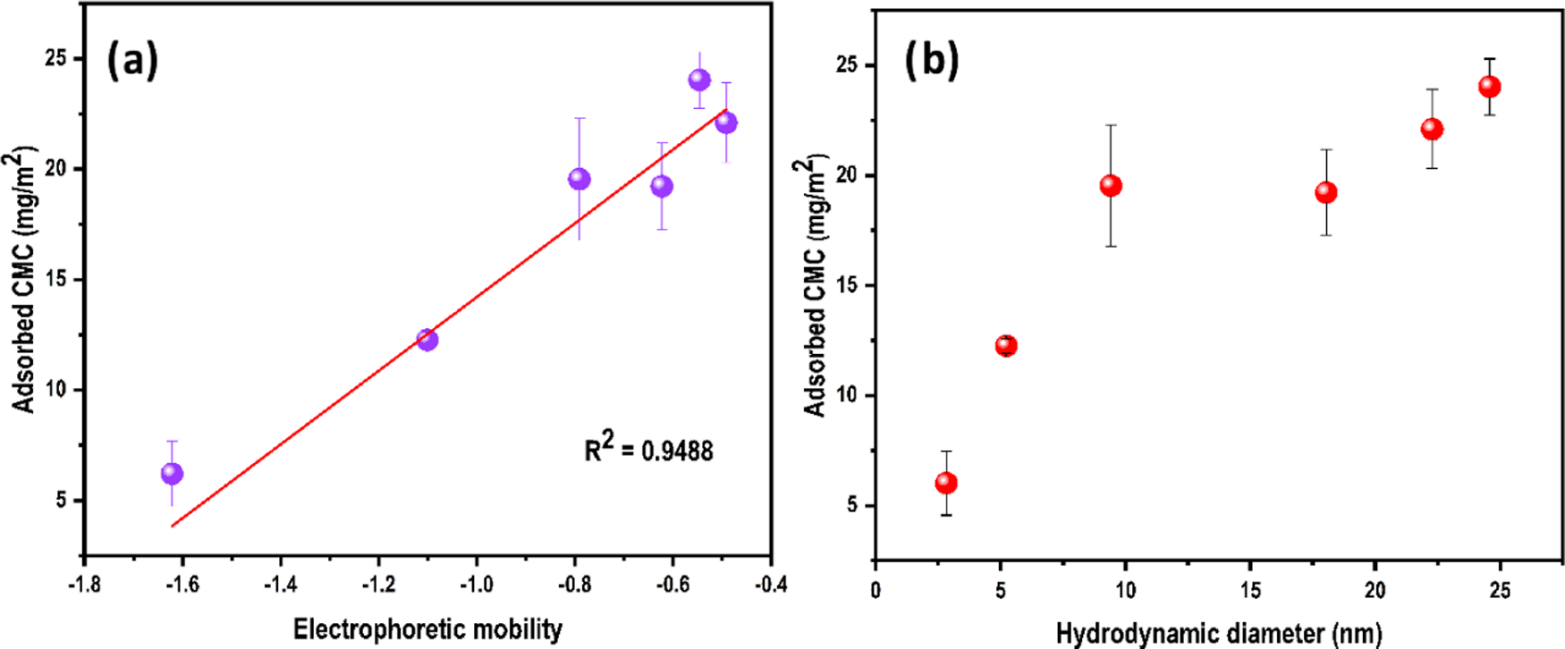
(a) Adsorbed CMC per unit area at different ionic strengths of CaCl_2_ plotted against electrophoretic mobility. (b) Hydrodynamic dynamic diameter at a corresponding concentration of CaCl_2_.

Figure 10a,b shows how the adsorbed CMC is related to the electrophoretic mobility and structural properties of the CMC in the presence of CaCl_2_, respectively. The mass of CMC adsorbed per unit area on cellulose increases with increases in both electrophoretic mobility and hydrodynamic diameter. However, when combining data in Figures 4 and 9, no such correlation could be observed between the ζ potential of the CNF surface and the adsorbed CMC (data not shown). From the observed ζ potential values, it is evident that even at an ionic strength of 10 mM, the charge on the surface becomes close to neutral. Further increase in ionic strength did not change the surface charge of the CNF appreciably. On the other hand, the CMC adsorbed per unit area increased with increased ionic strength up to 250 mM, meaning that Ca^2+^ ions have a profound effect on the solution side, which contributed to the adsorption. This explains the correlation between the CMC adsorbed and the hydrodynamic size. Moreover, screening negative charges and multichain association of CMC in the presence of CaCl_2_ reduces the polymer–solvent interaction, which could contribute to the driving force of adsorption. However, this might not be the sole driver of the adsorption of CMC onto cellulose. Recently, it has been suggested that the adsorption of hemicellulose on highly hydrated systems such as cellulose is driven by the entropy gain due to the release of structured water.^26,27^ Furthermore, a similar mechanism has been suggested for the adsorption of negatively charged PEDOT: PSS on the cellulose surface.^28^ To test this hypothesis in the case of CMC adsorption, we compared the adsorption of CMC from deionized water and deuterated water (D_2_O) at ionic strength 250 mM CaCl_2_, where the adsorption is expected to be driven by nonelectrostatic forces, according to Fleer et al.^44^

**Figure 11:**
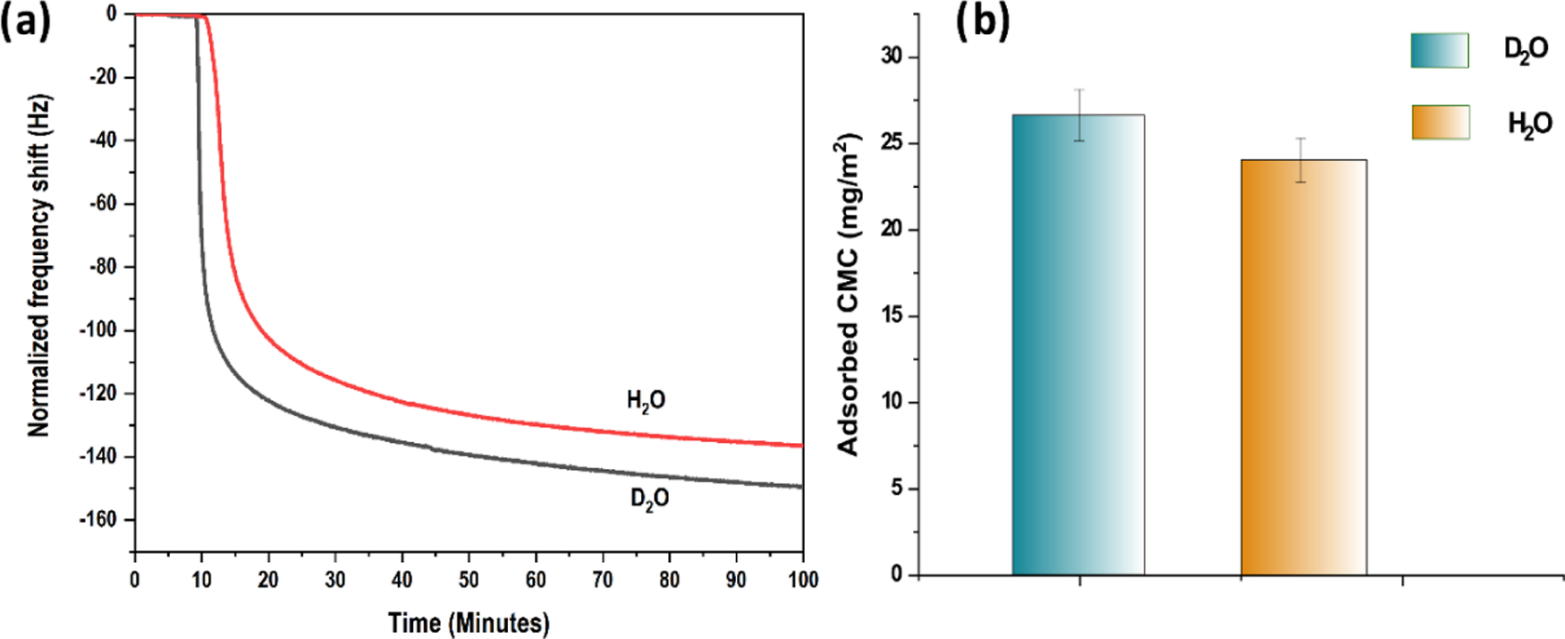
(a) Representative QCM-D frequency curve of adsorption of CMC on cellulose from D_2_O and H_2_O. (b) Adsorbed CMC calculated using Johannsmann’s model.

The observed change in frequency shift indicates that CMC adsorption is more favored from D_2_O than H_2_O. Thus, even though D_2_O and H_2_O are often considered similar liquids, there is a significant difference in their physicochemical properties stemming from differences in their intermolecular forces.^48^ D_2_O forms a stronger and higher average number of hydrogen bonds (10% more) compared to water.^49−51^ Thus, it is a more structured liquid than H_2_O.^52^ Therefore, when CMC is adsorbed from D_2_O, the entropy gain due to the release of structured D_2_O molecules from the cellulose surface would be more significant and result in high adsorption than CMC adsorbed from H_2_O, as is seen in Figure 11. It has also been suggested that in D_2_O, hydrophobic interactions are more significant.^53^ Seminal work by Laine et al. has shown that the adsorption of CMC increases with an increase in temperature, which also supports the claim of the entropy-driven mechanism of CMC adsorption.^4,27^

## 4 Conclusions

Previous investigations of the adsorption of CMC on cellulose, negatively charged polyelectrolytes in general, have overlooked the changes that occur in the properties of the polymer solution in the presence of multivalent ions, such as Ca^2+^. In this study, the effect divalent ions have on the adsorption of CMC onto cellulose has been studied from the perspectives that the adsorption process is multifaceted and can be influenced by the characteristics of the cellulose surface and the solution. Dynamic light scattering experiments performed on CMC solutions with varying concentrations of CaCl_2_ revealed the multichain association of CMC in the presence of CaCl_2_, which increased the hydrodynamic size. Furthermore, a correlation has been observed between the hydrodynamic diameter of CMCs in different concentrations of CaCl_2_ and the amount of CMC adsorbed in the corresponding concentrations of CaCl_2_. This suggests that the adsorption of CMCs at high concentrations of CaCl_2_ is mainly governed by structural changes in the CMC, which effectively reduce polymer–solvent interactions and thus contribute to the driving force of adsorption. Furthermore, we provided an experimental evidence for the entropy-driven adsorption of CMC on cellulose by comparing the adsorption of CMC from D_2_O and H_2_O.

## Supporting Information Available

QCM-D curve for D_2_O–H_2_O exchange studies on a bare sensor and the CNF thin film (third overtone) (Figure S1); height profile of CNF model films (Figure S2); and electrophoretic mobility and hydrodynamic diameter and adsorbed CMC per unit area of the cellulose surface as a function of the charge ratio between Ca^2+^ and CMC (Figures S3 and S4) (PDF)
