# Supplementary material for: Calcium Ion-Induced Structural Changes in Carboxymethylcellulose Solutions and Their Effects on Adsorption on Cellulose Surfaces
Source: Biomacromolecules. 2021 Dec 22;23(1):47–56. doi: 10.1021/acs.biomac.1c00895 (PMC8753602; doi:10.1021/acs.biomac.1c00895)
Supplement: Supplementary file 1 — bm1c00895_si_001.pdf [file bm1c00895_si_001.pdf]

Supporting information

Calcium ion-induced structural changes in carboxymethylcellulose solutions and their effects on adsorption on cellulose surfaces

Vishnu Arumughan[1,2], Tiina Nypelö[1,3], Merima Hasani[1,2,3] & Anette Larsson[1,2,3,4*]*

1. Department of Chemistry and Chemical Engineering, Chalmers University of Technology, Gothenburg, Sweden
2. AvanCell, Chalmers University of Technology, Gothenburg, Sweden
3. Wallenberg Wood Science Center, Chalmers University of Technology, Gothenburg, Sweden
4. FibRe – Centre for Lignocellulose-based Thermoplastics, Department of Chemistry and Chemical Engineering, Chalmers University of Technology, SE-412 96, Gothenburg, Sweden

[*] Corresponding author at Department of Chemistry and Chemical Engineering, Chalmers University of Technology, Gothenburg, Sweden

anette.larsson@chalmers.se (Anette Larsson) , vishnu.arumughan@chalmers.se (Vishnu Arumughan)

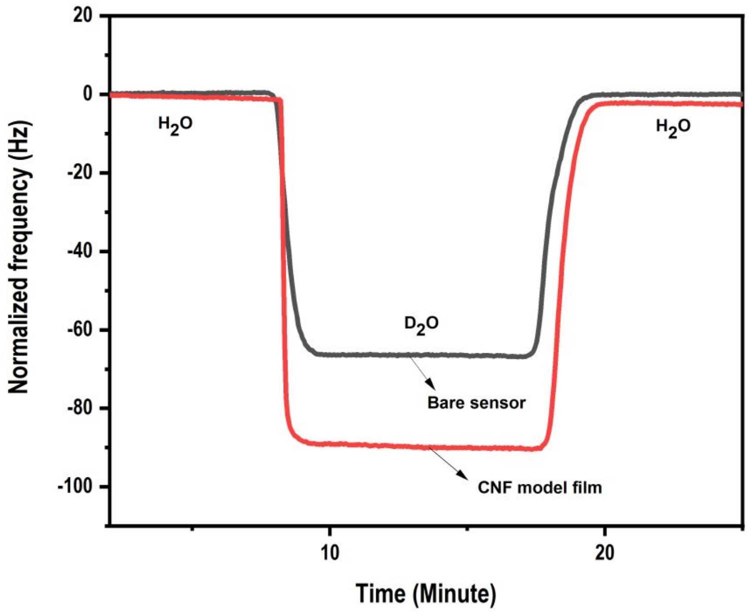

Figure S1. A representative QCM frequency change of D_2O- H_2O exchange studies on a bare sensor and CNF thin film (3rd Over tone)

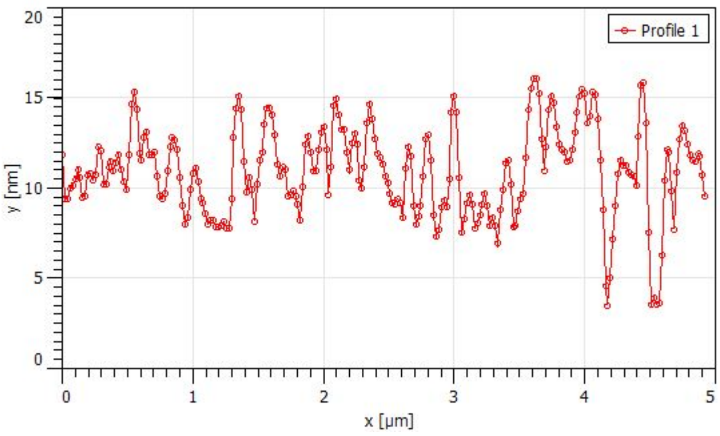

Figure S2. A typical height profile of CNF model film along a horizontal line.

The charge density of the CMC used in this study was determined to be 3.8 meq/g (determined using polyelectrolyte titrations). The concentration of the CMC in the adsorbing solution was 0.02 g/100 ml; therefore, we get around 80 µeq/100 ml. The ratio was calculated by dividing the number of charges from Ca^{2+} in 100 ml 80 µeq/100 ml.

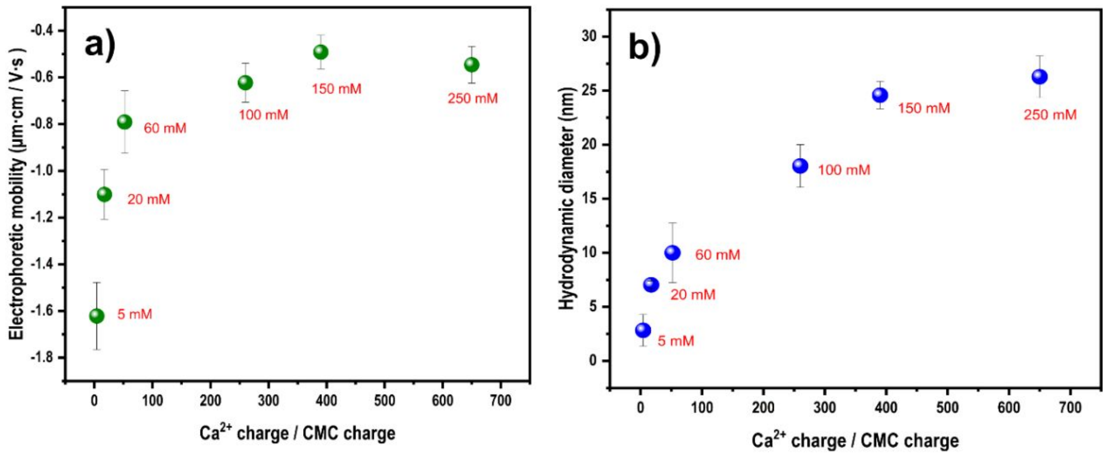

Figure S3. Electrophoretic mobility and hydrodynamic diameter of CMC as a function of charge ratio between Ca^{2+} and CMC

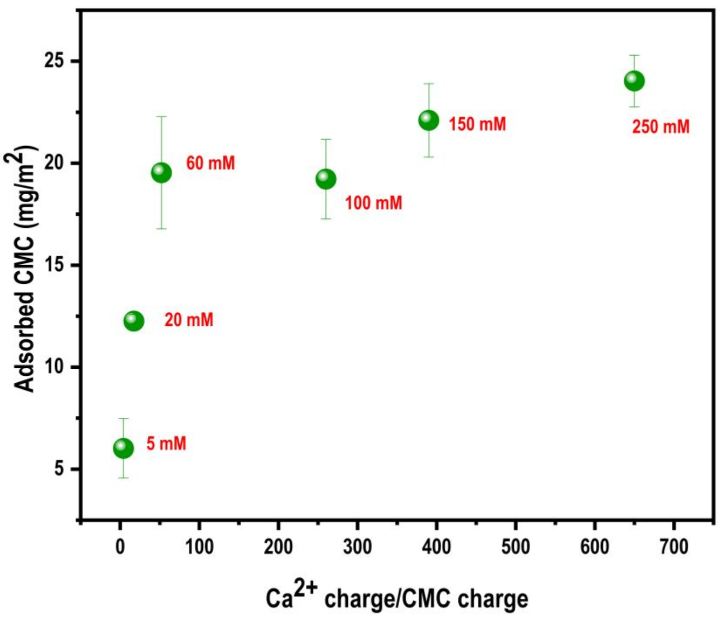

Figure S4. Adsorbed CMC per unit area as a function of charge ratio between Ca^{2+} and CMC

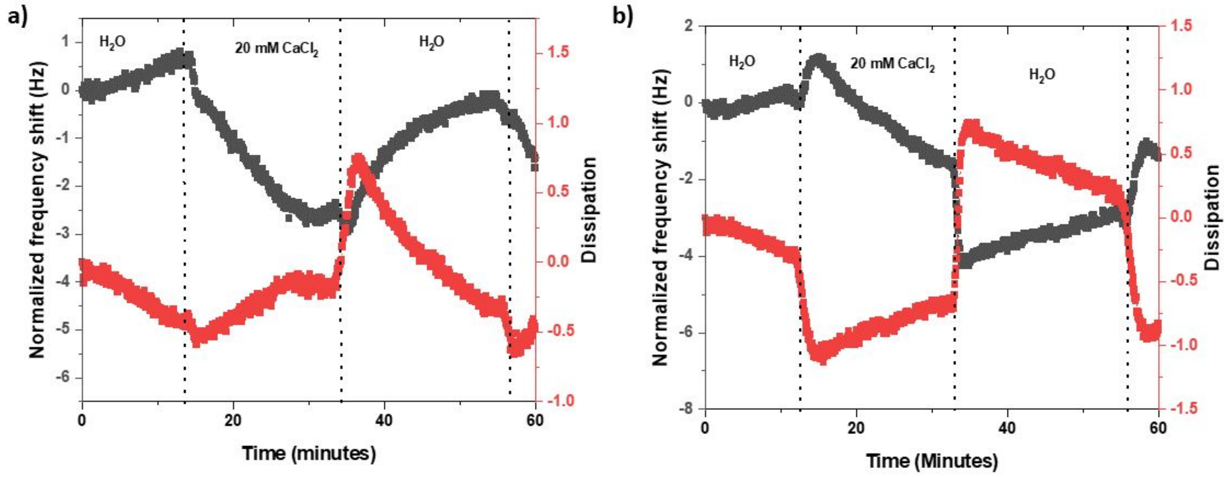

Figure S5 Reference measurements of swelling-deswelling studies a) SiO_2 coated bare sensor b) CNF coated sensor.

In the a) above, it is evident that the frequency shift to a more negative value when switched to 20 mM $CaCl_2$. However, this change is relatively low. Usually, a change of 1-2 Hz is considered within the error limit. In the case of CNF film, the behavior was the opposite, where the frequency shifted to a more positive value when switched to 20 mM $CaCl_2$, which could be due to the de-swelling of CNF film. The change is also within the error limit, indicating that the data are shown in the manuscript (figure 8) is valid.
